# Sexual Transmission of XMRV: A Potential Infection Route

**DOI:** 10.1155/2011/965689

**Published:** 2011-07-24

**Authors:** Prachi Sharma, Kenneth A. Rogers, Suganthi Suppiah, Ross J. Molinaro, Nattawat Onlamoon, John Hackett, Gerald Schochetman, Eric A. Klein, Robert H. Silverman, François Villinger

**Affiliations:** ^1^Division of Pathology, Yerkes National Primate Research Center, Emory University, Atlanta, GA 30329, USA; ^2^Department of Pathology and Laboratory Medicine, Emory University School of Medicine, Atlanta, GA 30329, USA; ^3^Office for Research and Development, Faculty of Medicine, Siriraj Hospital, Mahidol University, Bangkok, Bangkok 10700, Thailand; ^4^Abbott Diagnostics, Emerging Pathogens and Virus Discovery, Abbott Park, IL 60064, USA; ^5^Glickman Urological and Kidney Institute and LRI, Cleveland Clinic Foundation, Cleveland, OH 44195, USA; ^6^Lerner Research Institute, Cleveland Clinic Foundation, Cleveland, OH 44195, USA

## Abstract

Although XMRV dissemination in humans is a matter of debate, the prostate of select patients seem to harbor XMRV, which raises questions about its potential route of transmission. We established a model of infection in rhesus macaques inoculated with XMRV. In spite of the intravenous inoculation, all infected macaques exhibited readily detectable XMRV signal in the reproductive tract of all 4 males and 1 female during both acute and chronic infection stages. XMRV showed explosive growth in the acini of prostate during acute but not chronic infection. In seminal vesicles, epididymis, and testes, XMRV protein production was detected throughout infection in interstitial or epithelial cells. In the female monkey, epithelial cells in the cervix and vagina were also positive for XMRV gag. The ready detection of XMRV in the reproductive tract of male and female macaques infected intravenously suggests the potential for sexual transmission for XMRV.

## 1. Introduction

Xenotropic murine leukemia virus-related retrovirus (XMRV), a gammaretrovirus, was initially discovered in a study that used prostate carcinoma tissues and later in a study that used blood of chronic fatigue syndrome patients [1, 2], although other labs have been unable to detect XMRV in such patients [3] or have suggested that most of the findings based on nucleic amplification techniques were secondary to contamination with mouse DNA [4–7]. Moreover the link to chronic fatigue has recently been seriously questioned [8]. However, the detection of XMRV in select prostate cancers was not all based on methods using nucleic acid amplification, and while the etiological role of XMRV infection for cancer remains uncertain, XMRV represents a novel gamma retrovirus capable of infecting several human host cell lines [9], and hence, a potential zoonotic agent. In fact, infection of humans with this virus might have resulted from a zoonotic transmission from mouse to man [1] similar to the transspecies transmission reported with the Koala retrovirus, a gammaretrovirus closely related to XMRV [10]. Though both cell-associated and cell-free transmission of XMRV have been reported [2] and there is indirect evidence for potential respiratory [11] or sexual [12] transmission, the exact route or mechanism of transmission still remains unresolved. To better understand the XMRV pathogenesis, our lab recently established an animal model of XMRV infection using rhesus macaques. Results of this study have recently been published, demonstrating unequivocally that XMRV is infectious for primates, inducing a persistent infection which, given the right context, may be reactivated in vivo [13]. During the histological analyses of these animals, it was realized that in spite of the generalized infection, XMRV appeared to show a predilection for tissues and organs of the reproductive tract in this model, suggesting the potential for sexual transmission.

## 2. Materials and Methods

### 2.1. Animals

Nine adult healthy rhesus macaques (5 infected—4 males and 1 female and 4 controls—3 males and 1 female) of Indian origin with ages ranging from 5–17 years were used for this study. These macaques were housed at the Yerkes National Primate Research Center at Emory University under BSL-2+ housing conditions and maintained in accordance with the instructions of the Committee on the Care and Use of Laboratory Animals of the Institute of Laboratory Animal Resources, National Research Council, and the U.S. Public Health Service (PHS) Guidelines, *Guide for the Care and Use of Laboratory Animals*.

### 2.2. XMRV Inoculation

Five monkeys were inoculated with 3.6 × 10^6^ TCID_50_ of XMRV grown in DU145 prostate cancer cells delivered intravenously [13]. Euthanasia and necropsies of the first 3 monkeys were performed at days 6, 7 (acute infection, RLm-1 and ROu-4, resp.), and 144 pi (RLq-10) after a single-XMRV infection. The last 2 animals (RYl-10 and RIm-10) were reinoculated at day 158 after infection (pi) with 3.6 × 10^6^ TCID_50 _ of sucrose-purified XMRV (Advanced Biotechnologies, Inc., Columbia, Md, USA) again delivered IV. These 2 monkeys were euthanized at day 291/133 pi (after primary/secondary infection, resp.). At necropsy, reproductive tissue from the males (prostate, testes, seminal vesicle, and epididymis) and females (cervix and vagina) were collected in 10% neutral buffered formalin and fixed for 24 hours. Control animals (three males, one female) were not infected with XMRV, but housed and cared for in similar accommodations, but not in the same rooms. Three of the controls were sacrificed for noninfectious reasons, and one (male) was chronically infected with simian immunodeficiency, but without clinical progression to AIDS.

### 2.3. Immunohistochemistry

The formalin-fixed paraffin-embedded tissue was sectioned at 4 *μ*M thickness and slides prepared. After deparaffinization, the slides were rehydrated and antigen retrieval done by microwave treatment after quenching of endogenous peroxydases. The sections were incubated with a 1 : 100 dilution of a rat anti-SFFV antibody cross-reactive to XMRV [1] followed by biotinylated antirat polyclonal antibody and the ABC reagent (Vector Laboratories). Virus was detected by the development of the chromogenic substrate 3,3′-diaminobenzidine (Dako) and counterstained with hematoxylin. Each run was performed with relevant negative and positive controls. Slides were read using an Olympus BX-41 light microscope. Fluorescent in situ hybridization (FISH) to XMRV nucleic acids was performed as described previously [1, 13]. Though some recent reports that have suggested XMRV detection using RT-PCR in specimens collected from human patients may be the result of laboratory contamination [4–7], the data generated in our study did not rely on amplification techniques involving PCR. 

The techniques used here (IHC and FISH) were not susceptible to contamination errors, our laboratory does not work with rodent tissues, and while tissue collections from each animal were done at different times, embedding and slides preparation were processed as a batch.

## 3. Results and Discussion

A detailed analysis of the XMRV infection, viral dissemination, and antibody responses in these macaques is published elsewhere [13]. Here, our analyses focused on the viral distribution in the reproductive tract and its potential implication for sexual transmission. Briefly, none of the infected animals showed any obvious clinical symptoms for the entire 9 months of followup, based on activity levels, food intake, hematological, or serum chemistry findings. The infected animals were sacrificed at different times after infection to obtain tissues representative of the acute and chronic virus dissemination with clear separation from one another. Control and XMRV-infected tissues were, however, embedded and sectioned in parallel, and assayed together as batches. All tissues were Lymphoid organs like spleen, lymph nodes, and the gastrointestinal lamina propria and nonlymphoid organs like lung, brain, liver, and bone marrow contained cells positive for XMRV by IHC and/or FISH for which semiquantitative analysis was performed [13]. Of note, several tissues such as CNS, heart, adrenal gland, gall bladder, kidney, and urinary bladder were negative by IHC with only a rare single FISH signal [13]. In contrast, there was an absence of signal all organs of uninfected control rhesus macaques run in the same batches as samples from XMRV infected monkeys.

**Figure 1:**
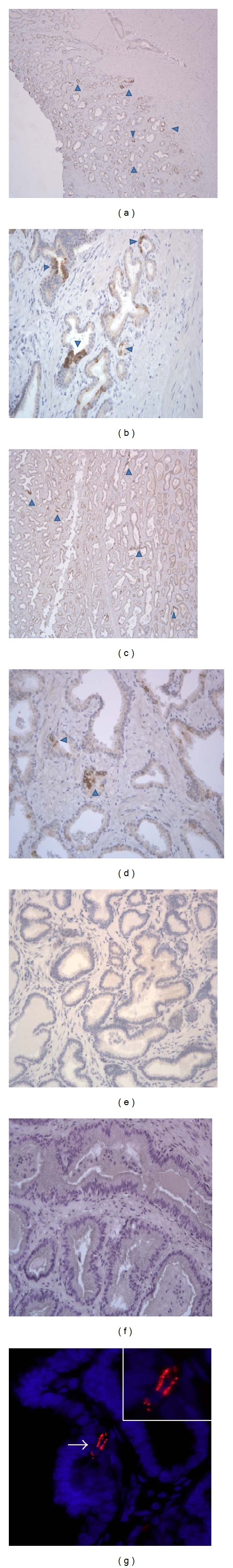
Detection of XMRV gag in the prostates of two acutely infected macaques RLm-1 ((a, b), day 6 pi) and ROu-4 ((c, d), day 7 pi); one chronically infected macaque RLq-10 ((e, f), day 144 pi); control macaque RPd-7 (f). Immunohistochemistry ((a)–(f)). Magnification, ×40 ((a, c)), ×200 ((b, d, e, and f)). FISH (g), magnification, ×600, ×1000.

No significant gross or histologic lesions were observed in the reproductive tract of all animals except for the presence of mononuclear cell infiltrates in prostate tissues [13], although such findings are not uncommon in age matched controls. During acute XMRV infection (days 6-7 pi) extensive foci of XMRV, positive acinar epithelial cells were detected in the prostate of these monkeys. (Figures 1(a) and 1(d)). Figures 1(a) and 1(c) (arrows) show an overview of the multiple foci of positively staining glandular epithelial cells in the prostate of the two acutely infected animals. The presence of XMRV in the epithelial cells of the prostate, with the surrounding stroma being negative during the acute infection, suggests cell-to-cell transmission in the glandular acini and also that prostate is a predilection site for the early XMRV infection. Though XMRV was no longer detected by IHC in the prostate during the chronic phase of infection (Figure 1(e)), similar to the control macaques (Figure 1(f)), XMRV nucleic acid signals were still observed using FISH (Figure 1(g)). This suggests a lack of replication, an unsuitable environment, or some form of active control of viral replication which remains to be determined. 

**Figure 2:**
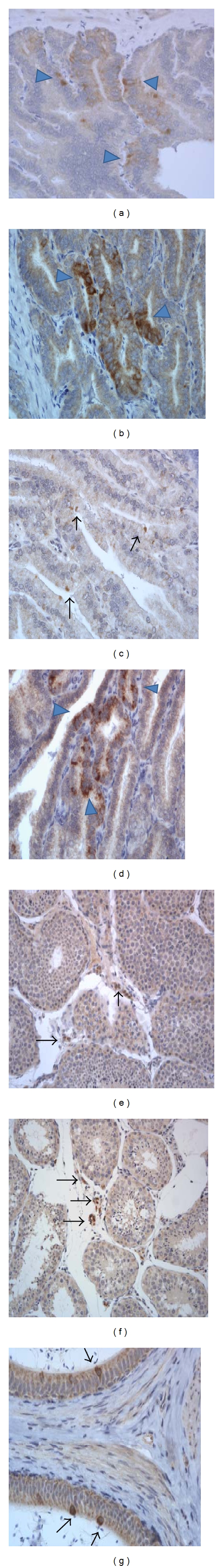
Detection of XMRV gag in the other reproductive tissues of the infected male macaques: seminal vesicle, ROu-4 ((a) day 7 pi), RLm-1 ((b) day 6 pi), RIl-10 ((c) day 291/133 pi), RLq-10 ((d) day 144 pi), testis, RIl-10 ((e) day 291/133 pi), testis, RLq-10 ((f) day 144 pi), epididymis, RLq-10 ((g) day 144 pi). Immunohistochemistry. Magnification, ×400 (a, b, c, d, g) and ×200 (e, f).

**Figure 3:**
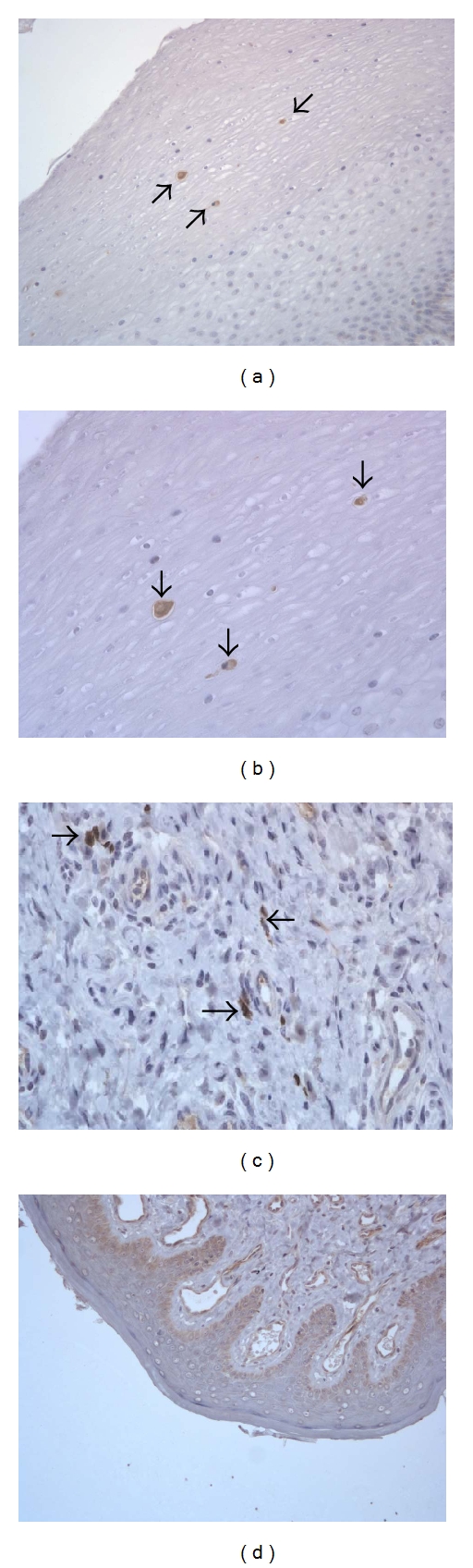
Detection of XMRV gag in the cervix (a, b) and vagina (c) of the female rhesus macaque (RYh-10, day 291/133 pi), and in the vagina of a control female rhesus macaque RFm-2 (d). Magnification, ×200 (a, d), ×400 (b, c).

Other tissues showing positive staining in the male-reproductive tract included seminal vesicles (Figure 2(a) and 2(d)) and testes of both acute (Figure 2(e)) and chronically (Figure 2(f)) infected animals. The epididymis of one chronically infected animal (Figure 2(g)) revealed frequent positive epithelial cells. Of interest, the morphology of the cells expressing XMRV protein varied from glandular epithelial cells in the prostate, seminal vesicle, and epididymis to the interstitial cells in the testes. Remarkably, even the reproductive tissue sampled from the only female monkey included in our study showed positive XMRV staining at 9 months after infection. Occasional mucosal epithelial cells in the cervical epithelium (Figures 3(a) and 3(b)) and submucosal cells, presumably fibroblasts, (Figure 3(c)) in the vaginal submucosa expressed XMRV gag protein. Viral protein detection was consistently negative in the reproductive tract of the control female rhesus macaque (Figure 3(d)). 

Our study used the intravenous mode of virus administration to ensure infection. But, despite using this route, the virus to our surprise rapidly concentrated in the lower reproductive tract of both male and female macaques, suggesting a potential for the sexual mode of transmission. Lending support to this hypothesis are previous reports showing the detection of XMRV RNA in human prostatic secretions [12], and the finding that XMRV replication is enhanced by androgens due to the presence of a glucocorticoid response element (GRE) [7]. Moreover, human semen or cationic amyloid fibrils, a degradation product from prostatic acid phosphatase, has been shown to promote the transmission of HIV and XMRV in vitro [12, 14, 15], suggesting another mechanism potentially facilitating mucosal transmission in vivo. Though recent reports have suggested that XMRV detection using RT-PCR in specimens collected from human patients may be the result of laboratory contamination [12], the data generated in our study relied on techniques (IHC and FISH) that did not include gene amplification and are therefore rather impervious to sample contamination. Moreover, negative controls on this study were consistently negative using these techniques. 

In conclusion, our study demonstrates XMRV protein expression in the reproductive tract of the experimentally infected rhesus macaques at all times after infection supporting the potential for sexual transmission of this virus.

##  Author Contributions

Eric A. Klien, John Hackett, Robert H. Silverman, and François Villinger designed the study. Prachi Sharma, Suganthi Suppiah, Ross J. Molinaro, Nattawat Onlamoon, Kenneth A. Rogers, and François Villinger performed the research and analyzed the data. Prachi Sharma wrote the paper.

## References

[1] Urisman A, Molinaro RJ, Fischer N (2006). Identification of a novel gammaretrovirus in prostate tumors of patients
homozygous for R462Q RNASEL variant. *PLoS Pathogens*.

[2] Lombardi VC, Ruscetti FW, Gupta JD (2009). Detection of an infectious retrovirus, XMRV, in blood cells of patients with
chronic fatigue syndrome. *Science*.

[3] Stoye JP, Silverman RH, Boucher CA, Le Grice SF (2010). The xenotropic murine leukemia virus-related retrovirus debate continues at
first international workshop. *Retrovirology*.

[4] Hue S, Gray ER, Gall A (2010). Disease-associated XMRV sequences are consistent with laboratory
contamination. *Retrovirology*.

[5] Oakes B, Tai AK, Cingoz O (2010). Contamination of human DNA samples with mouse DNA can lead to false detection
of XMRV-like sequences. *Retrovirology*.

[6] Robinson MJ, Erlwein OW, Kaye S (2010). Mouse DNA contamination in human tissue tested for XMRV. *Retrovirology*.

[7] Sato E, Furuta RA, Miyazawa T (2010). An endogenous murine leukemia viral genome contaminant in a commercial RT-PCR
Kit is amplified using standard primers for XMRV. *Retrovirology*.

[8] Shin CH, Bateman L, Schlaberg R (2011). Absence of XMRV and other murine leukemia virus-related viruses in
patients with
chronic fatigue syndrome. *The Journal of Virology*.

[9] Stieler K, Schulz C, Lavanya M, Aepfelbacher M, Stocking C, Fischer N (2010). Host range and cellular tropism of the human exogenous gammaretrovirus
XMRV. *Virology*.

[10] Tarlinton RE, Meers J, Young PR (2006). Retroviral invasion of the koala genome. *Nature*.

[11] Fischer N, Schulz C, Stieler K (2010). Xenotropic murine leukemia virus-related gammaretro virus in respiratory
tract. *Emerging Infectious Diseases*.

[12] Hong S, Klein EA, Das Gupta J (2009). Fibrils of prostatic acid phosphatase fragments boost infections with XMRV
(xenotropic murine leukemia virus-related virus), a human retrovirus associated with
prostate cancer. *The Journal of Virology*.

[13] Onlamoon N, Gupta JD, Sharma P (2011). Infection, viral dissemination and antibody responses of rhesus macaques
exposed to the human gammaretrovirus XMRV. *The Journal of Virology*.

[14] Münch J, Rücker E, Ständker L (2007). Semen-derived amyloid fibrils drastically enhance HIV infection. *Cell*.

[15] Roan NR, Münch J, Arhel N (2009). The cationic properties of SEVI underlie its ability to enhance human
immunodeficiency virus infection. *The Journal of Virology*.

